# High-Quality draft genome sequence of the *Lotus* spp*.* microsymbiont *Mesorhizobium loti* strain CJ3Sym

**DOI:** 10.1186/s40793-015-0049-2

**Published:** 2015-08-14

**Authors:** Wayne Reeve, John Sullivan, Clive Ronson, Rui Tian, Christine Munk, Cliff Han, T.B.K. Reddy, Rekha Seshadri, Tanja Woyke, Amrita Pati, Victor Markowitz, Natalia Ivanova, Nikos Kyrpides

**Affiliations:** Centre for Rhizobium Studies, Murdoch University, Perth, WA Australia; Department of Microbiology and Immunology, University of Otago, Dunedin, New Zealand; Los Alamos National Laboratory, Bioscience Division, Los Alamos, NM USA; DOE Joint Genome Institute, Walnut Creek, CA USA; Biological Data Management and Technology Center, Lawrence Berkeley National Laboratory, Berkeley, CA USA; Department of Biological Sciences, King Abdulaziz University, Jeddah, Saudi Arabia

**Keywords:** Root-nodule bacteria, Nitrogen fixation, Symbiosis, Alphaproteobacteria, GEBA-RNB

## Abstract

*Mesorhizobium loti* strain CJ3Sym was isolated in 1998 following transfer of the integrative and conjugative element ICE*Ml*Sym^R7A^, also known as the R7A symbiosis island, in a laboratory mating from the donor *M. loti* strain R7A to a nonsymbiotic recipient *Mesorhizobium* strain CJ3. Strain CJ3 was originally isolated from a field site in the Rocklands range in New Zealand in 1994. CJ3Sym is an aerobic, Gram-negative, non-spore-forming rod. This report reveals the genome of *M. loti* strain CJ3Sym currently comprises 70 scaffolds totaling 7,563,725 bp. The high-quality draft genome is arranged in 70 scaffolds of 71 contigs, contains 7,331 protein-coding genes and 70 RNA-only encoding genes, and is part of the GEBA-RNB project proposal.

**Electronic supplementary material:**

The online version of this article (doi:10.1186/s40793-015-0049-2) contains supplementary material, which is available to authorized users.

## Introduction

*Mesorhizobium loti* strain CJ3Sym was first described in work that showed that the symbiotic genes of *M. loti* strain R7A (a field reisolate of culture collection strain ICMP3153) were located on a large transmissible symbiosis island that could be transferred to nonsymbiotic mesorhizobia both in the laboratory and the environment [1, 2]. The symbiosis island was later classified as an integrative and conjugative element and renamed ICE*Ml*Sym^R7A^ [3]. CJ3Sym was derived from a nonsymbiotic *Mesorhizobium* strain CJ3 by transfer of the symbiosis island from R7A in a laboratory mating experiment. The CJ3Sym progenitor strain CJ3 was a nonsymbiotic *Mesorhizobium* strain that was isolated from the rhizosphere of a *Lotus corniculatus* L. bird's-foot trefoil cv. Grasslands Goldie (here after referred to as *Lotus corniculatus* cv. Grasslands Goldie) plant taken from a field site in the Rocklands range, Central Otago, New Zealand in 1994, near where ICE*Ml*Sym^R7A^ was discovered [4]. The study was initiated to locate nonsymbiotic rhizobia that were postulated to be the likely progenitors of the diverse symbiotic strains that had received the symbiosis island through horizontal gene transfer at the field site.

Seven strains (CJ1 to CJ7) which had a similar colony morphology to *M. loti*, but which could not nodulate *Lotus corniculatus* cv. Grasslands Goldie and lacked *nod* and *nif* genes were isolated. The strains were shown to be closely related to the diverse symbiotic strains from the site by RFLP analysis, whole genome DNA-DNA hybridization analysis, full 16S rRNA gene sequencing and multilocus enzyme electrophoresis. The seven strains fell into four genomic species of nonsymbiotic mesorhizobia with strains CJ3, CJ1, CJ4 and CJ6 belonging to the same genomic species as the diverse symbiotic isolates.

When strains CJ1 to CJ7 were characterized it was noticed that they grew poorly, and only formed microcolonies after prolonged incubation on defined G/RDM agar media, in comparison to growth on rich YMA media. Auxanographic analysis revealed that all 7 strains were auxotrophic for thiamin and biotin and all but CJ5 were auxotrophic for nicotinate. In contrast to CJ3, strain CJ3Sym is prototrophic for all three vitamins and consistent with this the genes required for their biosynthesis are located on ICE*Ml*Sym^R7A^ [5]. The CJ3Sym sequence confirms that these are the only operons for the biosynthesis of the three vitamins in the genome.

## Organism information

### Classification and features

**Fig. 1:**
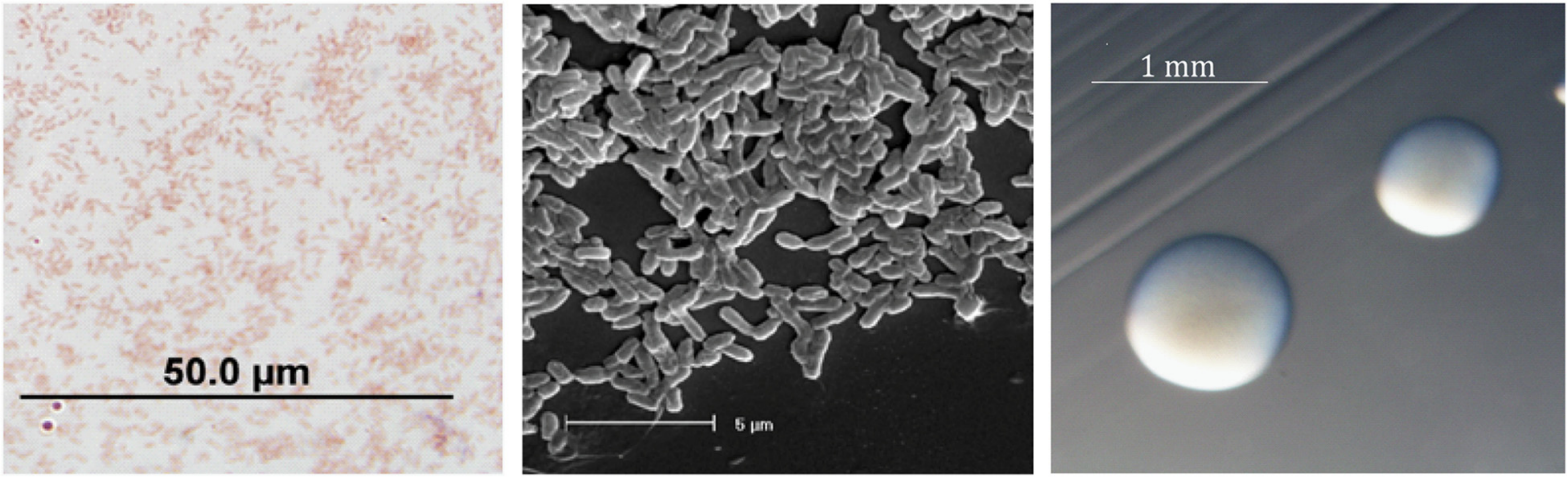
Images of *Mesorhizobium loti* strain CJ3Sym from a Gram stain (Left), using scanning electron microscopy (Center) and the appearance of colony morphology on ½LA (Right)

*Mesorhizobium loti* strain CJ3Sym is in the order *Rhizobiales* of the class *Alphaproteobacteria*. Cells are described as non-sporulating, Gram-negative, non-encapsulated, rods (Fig. 1 Left). The rod-shaped form varies in size with dimensions of 0.25-0.5 μm in width and 1.25-1.5 μm in length (Fig. 1 Left and Right). It forms 2 mm diameter colonies within 6 days and has a mean generation time of approximately 8 h when grown in TY broth at 28 °C [2]. Colonies on G/RDM agar [6] and half strength Lupin Agar (½LA) [7] are opaque, slightly domed, mucoid with smooth margins (Fig. 1 Right).

Strains of this organism are able to tolerate a pH range between 4 and 10. Carbon source utilization and fatty acid profiles of *M. loti* have been described previously [8–10]. Minimum Information about the Genome Sequence (MIGS) is provided in Table 1 and Additional file 1: Table S1.

**Table 1:** Classification and general features of *Mesorhizobium loti* strain CJ3Sym in accordance with the MIGS recommendations [30] published by the Genome Standards Consortium [31]

MIGS ID	Property	Term	Evidence code^a^
	Classification	Domain Bacteria	TAS [32]
		Phylum *Proteobacteria*	TAS [23, 33]
		Class *Alphaproteobacteria*	TAS [34]
		Order *Rhizobiales*	TAS [35]
		Family *Phyllobacteriaceae*	TAS [36]
		Genus *Mesorhizobium*	TAS [9]
		Species *Mesorhizobium loti*	TAS [8]
		Strain CJ3Sym	TAS [2]
	Gram stain	Negative	IDA
	Cell shape	Rod	IDA
	Motility	Motile	IDA
	Sporulation	non-sporulating	NAS
	Temperature range	Mesophile	NAS
	Optimum temperature	28 °C	NAS
	pH range; Optimum	Unknown	NAS
	Carbon source	various	TAS [9]
	Energy source	chemoorganotroph	TAS [9]
MIGS-6	Habitat	Soil, root nodule, host	TAS [8]
MIGS-6.3	Salinity	Unknown	NAS
MIGS-22	Oxygen requirement	Aerobic	TAS [8]
MIGS-15	Biotic relationship	Free living, Symbiotic	TAS [8]
MIGS-14	Pathogenicity	None	NAS
	Biosafety level	1	TAS [37]
	Isolation	Isolated following transfer of ICE*Ml*Sym^R7A^ from the donor *M. loti* strain R7A to a nonsymbiotic recipient *Mesorhizobium* strain CJ3 in a laboratory mating	TAS [2]
MIGS-4	Geographic location	Dunedin, Otago, NZ	TAS [2]
MIGS-5	Isolation date	1998	TAS
MIGS-4.1	Latitude	-45.864179	TAS [2]
MIGS-4.2	Longitude	170.512551	TAS [2]
MIGS-4.3	Depth	5-10 cm	IDA
MIGS-4.4	Altitude	50 m	IDA

^a^Evidence codes – *IDA* Inferred from Direct Assay, *TAS* Traceable Author Statement (i.e., a direct report exists in the literature), *NAS* Non-traceable Author Statement (i.e., not directly observed for the living, isolated sample, but based on a generally accepted property for the species, or anecdotal evidence). Evidence codes are from the Gene Ontology project [38, 39]

**Fig. 2:**
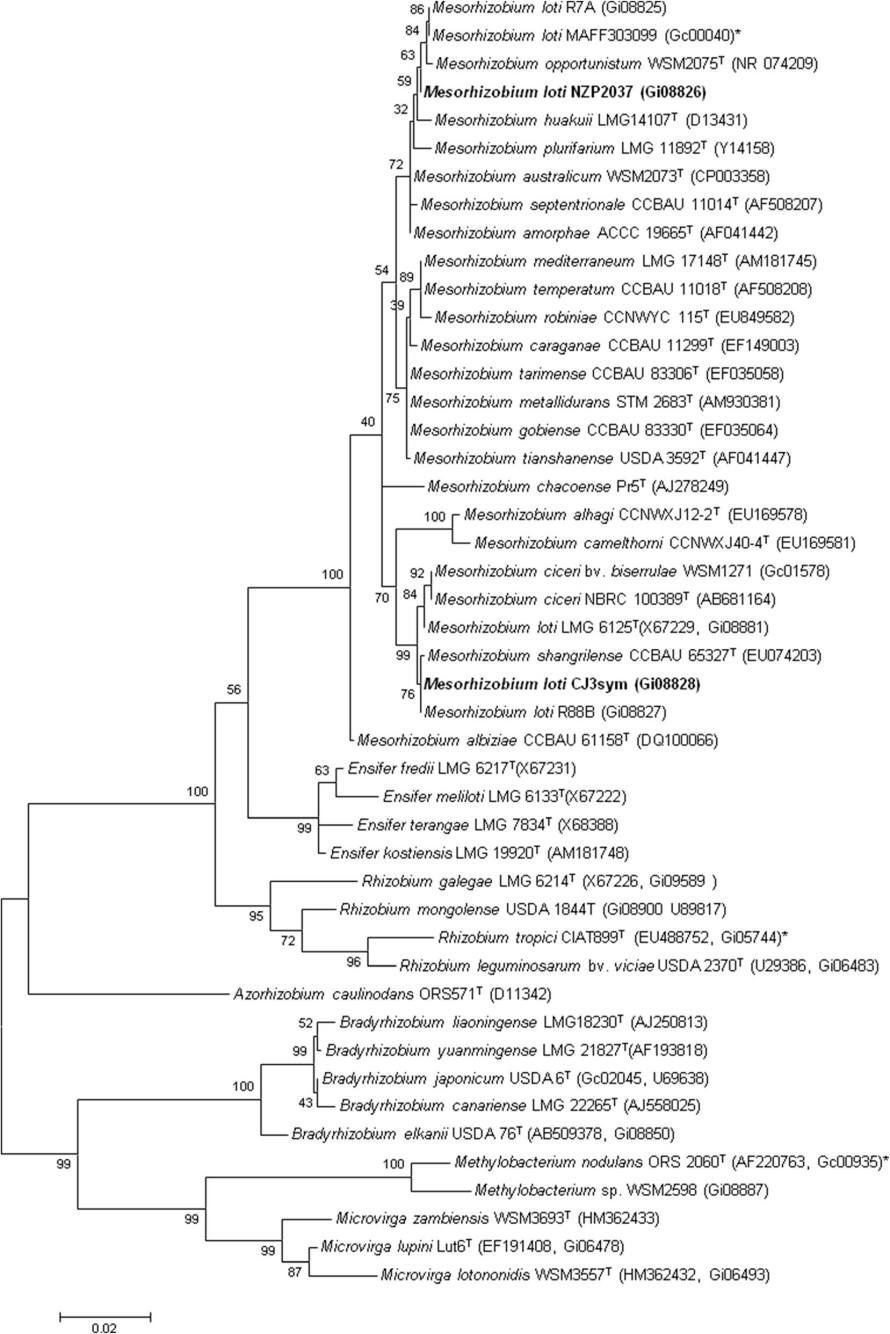
Phylogenetic tree showing the relationships of *Mesorhizobium loti* CJ3Sym with other root nodule bacteria based on aligned sequences of the 16S rRNA gene (1,290 bp internal region). All sites were informative and there were no gap-containing sites. Phylogenetic analyses were performed using MEGA [40], version 5. The tree was built using the Maximum-Likelihood method with the General Time Reversible model [41]. Bootstrap analysis [42] with 500 replicates was performed to assess the support of the clusters. Type strains are indicated with a superscript T. Brackets after the strain name contain a DNA database accession number and/or a GOLD ID (beginning with the prefix G) for a sequencing project registered in GOLD [43]. Published genomes are indicated with an asterisk

Figure 2 shows the phylogenetic neighborhood of *M. loti* strain CJ3Sym in a 16S rRNA gene sequence based tree. This strain has 99.8 % (1,364/1,366 bp) 16S rRNA gene sequence identity to *M. loti* R88B (GOLD ID: Gi08827) and 99.6 % sequence identity (1,361/1,366 bp) to *M. australicum* WSM2073 (GOLD ID: Gc02468). *M. loti* strain R88B is a diverse symbiotic strain isolated from the same field site as CJ3Sym, confirming the close relationship between symbiotic and nonsymbiotic mesorhizobia isolated from the site. It is interesting to note that both of these strains cluster with *Mesorhizobium shangrilense**,* several *Mesorhizobium ciceri* strains and the type *M. loti* strain LMG 6125 (NZP2213) whereas *M. loti* strains R7A, NZP2037 and MAFF303099 form a separate cluster that shares only 98 % 16S rRNA gene sequence identity with CJ3Sym and R88B.

#### Symbiotaxonomy

*Mesorhizobium* sp. strain CJ3Sym was isolated from a laboratory mating experiment in which ICEMlSym^R7A^ was transferred from the donor strain R7A to the nonsymbiotic *Mesorhizobium* strain CJ3 [2]. The nonsymbiont strain CJ3 was isolated from the rhizosphere of a *Lotus corniculatus* cv. Grasslands Goldie plant located at a field site that was an undeveloped tussock (*Festuca novae-zealandiae* and *Chionochloa rigida*) grassland located at an elevation of 885 m in Lammermoor, the Rocklands range, Otago, New Zealand in 1994 [4]. The soil was a dark brown silt loam with an acid pH (4.9) and a low (0.28 %) total nitrogen content [11]. CJ3 existed as a soil saprophyte that lacked symbiotic DNA. CJ3Sym forms effective nodules on *L. corniculatus* cv. Grasslands Goldie but has not yet been tested on any other *Lotus* species or ecotypes.

## Genome sequencing information

### Genome project history

This organism was selected for sequencing on the basis of its environmental and agricultural relevance to issues in global carbon cycling, alternative energy production, and biogeochemical importance, and is part of the Genomic Encyclopedia of Bacteria and Archaea, Root Nodulating Bacteria project at the U.S. Department of Energy, Joint Genome Institute. The genome project is deposited in the Genomes OnLine Database [12] and a high-quality permanent draft genome sequence in IMG [13]. Sequencing, finishing and annotation were performed by the JGI using state of the art sequencing technology [14]. A summary of the project information is shown in Table 2.

**Table 2:** Project information

MIGS ID	Property	Term
MIGS-31	Finishing quality	High-quality permanent draft
MIGS-28	Libraries used	One Illumina fragment library
MIGS-29	Sequencing platforms	Illumina HiSeq2000 technology
MIGS-31.2	Fold coverage	Illumina: 522x
MIGS-30	Assemblers	Velvet version 1.1.04; Allpaths-LG version r41043
MIGS-32	Gene calling methods	Prodigal 1.4, GenePRIMP
	Locus Tag	A3A9
	GenBank ID	AXAL00000000
	GenBank date of Relase	September 30, 2013
	GOLD ID	Gp0010090
	BIOPROJECT	PRJNA165305
MIGS-13	Source Material Identifier	CJ3Sym
	Project relevance	Symbiotic nitrogen fixation, agriculture

### Growth conditions and genomic DNA preparation

*M. loti* strain CJ3Sym was grown to mid logarithmic phase in TY rich medium [15] on a gyratory shaker at 28 °C. DNA was isolated from 60 mL of cells using a CTAB (Cetyl trimethyl ammonium bromide) bacterial genomic DNA isolation method [16]

### Genome sequencing and assembly

The draft genome of *M. loti* CJ3Sym was generated at the DOE Joint Genome Institute using Illumina technology [17]. An Illumina standard shotgun library was constructed and sequenced using the Illumina HiSeq 2000 platform, which generated 26,326,824 reads totaling 3,949 Mbp.

All general aspects of library construction and sequencing performed at the JGI can be found at the JGI’s web site [18]. All raw Illumina sequence data was passed through DUK, a filtering program developed at JGI, which removes known Illumina sequencing and library preparation artifacts (Mingkun L, Copeland A, Han J, Unpublished). The following steps were then performed for assembly: (1) filtered Illumina reads were assembled using Velvet [19] (version 1.1.04), (2) 1–3 Kbp simulated paired end reads were created from Velvet contigs using wgsim [20], (3) Illumina reads were assembled with simulated read pairs using Allpaths–LG [21] (version r41043). Parameters for assembly steps were: 1) Velvet --v --s 51 --e 71 --i 4 --t 1 --f "-shortPaired -fastq $FASTQ" --o "-ins_length 250 -min_contig_lgth 500"), 2) wgsim (−e 0–1 100–2 100 -r 0 -R 0 -X 0), 3) Allpaths–LG (STD_1,project,assembly,fragment,1,200,35,,,inward,0,0.

SIMREADS,project,assembly,jumping,1,,,3000,300,inward,0,0). The final draft assembly contained 71 contigs in 70 scaffolds. The total size of the genome is 7.6 Mbp and the final assembly is based on 3,949 Mbp of Illumina data, which provides an average of 522x coverage of the genome.

### Genome annotation

Genes were identified using Prodigal [22] as part of the DOE-JGI genome annotation pipeline [23], followed by a round of manual curation using the JGI GenePrimp pipeline [24]. The predicted CDSs were translated and used to search the National Center for Biotechnology Information non-redundant database, UniProt, TIGRFam, Pfam, KEGG, COG, and InterPro databases. The tRNAScanSE tool [25] was used to find tRNA genes, whereas ribosomal RNA genes were found by searches against models of the ribosomal RNA genes built from SILVA [26]. Other non–coding RNAs such as the RNA components of the protein secretion complex and the RNase P were identified by searching the genome for the corresponding Rfam profiles using INFERNAL [27]. Additional gene prediction analysis and manual functional annotation was performed within the Integrated Microbial Genomes-Expert Review (IMG-ER) system [28].

## Genome properties

The genome is 7,563,725 nucleotides with 62.15 % GC content (Table 3) and is comprised of a single scaffold. From a total of 7,401 genes, 7,331 were protein encoding and 70 RNA-only encoding genes. The majority of genes (76.76 %) were assigned a putative function whilst the remaining genes were annotated as hypothetical. The distribution of genes into COGs functional categories is presented in Table 4.

**Table 3:** Genome statistics

Attribute	Value	% of Total
Genome size (bp)	7,563,725	100.00
DNA coding (bp)	6,613,638	87.44
DNA G + C (bp)	4,700,964	62.15
DNA scaffolds	70	
Total genes	7,401	100.00
Protein-coding genes	7,331	99.05
RNA genes	70	0.95
Pseudo genes	0	0.00
Genes in internal biosynthetic clusters	478	6.46
Genes with function prediction	5,681	76.76
Genes assigned to COGs	5,074	68.56
Genes assigned Pfam domains	5,960	80.53
Genes with signal peptides	649	8.77
Genes coding transmembrane helices	1,688	22.81
CRISPR repeats	1

**Table 4:** Number genes associated with general COG functional categories

Code	Value	% of total (5,809)	COG Category
J	234	4.03	Translation, ribosomal structure and biogenesis
A	0	0.00	RNA processing and modification
K	526	9.05	Transcription
L	139	2.39	Replication, recombination and repair
B	5	0.09	Chromatin structure and dynamics
D	33	0.57	Cell cycle control, Cell division, chromosome partitioning
V	124	2.13	Defense mechanisms
T	216	3.72	Signal transduction mechanisms
M	309	5.32	Cell wall/membrane/envelope biogenesis
N	46	0.79	Cell motility
W	32	0.55	Extracellular structures
U	106	1.82	Intracellular trafficking, secretion, and vesicular transport
O	205	3.53	Posttranslational modification, protein turnover, chaperones
C	319	5.49	Energy production and conversion
G	519	8.93	Carbohydrate transport and metabolism
E	736	12.67	Amino acid transport and metabolism
F	102	1.76	Nucleotide transport and metabolism
H	274	4.72	Coenzyme transport and metabolism
I	282	4.85	Lipid transport and metabolism
P	286	4.92	Inorganic ion transport and metabolism
Q	225	3.87	Secondary metabolite biosynthesis, transport and catabolism
R	657	11.31	General function prediction only
S	383	6.59	Function unknown
-	2,327	31.44	Not in COGS

## Conclusions

The *M. loti* strain CJ3Sym genome was completed to the stage where 70 scaffolds comprising 71 contigs and 7.56 Mb were obtained. A total of 7,401 genes were annotated. It is likely that the genome consists of a single chromosome and a single plasmid; however further assembly is required to confirm this*.* CJ3Sym is a strain that was derived from nonsymbiotic *Mesorhizobium* strain CJ3 by transfer of the symbiosis island ICE*Ml*Sym^R7A^ from *M. loti* strain R7A in a laboratory mating experiment [2]. After the discovery of diverse *M. loti* strains containing ICE*Ml*Sym^R7A^ at a New Zealand field site, a second adjacent field site was established and sampled to identify nonsymbiotic mesorhizobia that were the likely progenitors of the diverse symbiotic strains. Strain CJ3 was one of seven non-symbiotic *Mesorhizobium* strains isolated from the rhizosphere of *Lotus corniculatus* cv. Grasslands Goldie plants and one of the four that belonged to the same genomic species as the diverse symbiotic isolates that contained ICE*Ml*Sym^R7A^ [4]. The genome of CJ3Sym is likely to contain a plasmid, as scaffold 17.18 contains a *trb* gene cluster (Locus tags 05060–05072 coordinates 16432–26076) and *traG* (locus tag 05072 coordinates 26704–28695) highly similar to genes on the *M. loti* strain MAFF303099 pMlb plasmid [29]. The same scaffold also contains likely plasmid replication genes.

## Additional file

Additional file 1: Table S1. Associated MIGS record for *Mesorhizobium loti* CJ3Sym. (DOC 73 kb)

## Abbreviations

GEBA-RNB: Genomic Encyclopedia for Bacteria and Archaea-Root Nodule Bacteria
JGI: Joint Genome Institute
½LA: half strength Lupin Agar
TY: Tryptone Yeast
YMA: Yeast Mannitol Agar
CTAB: Cetyl Trimethyl Ammonium Bromide
