# Supplementary material for: High-Quality draft genome sequence of the Lotus spp. microsymbiont Mesorhizobium loti strain CJ3Sym
Source: Stand Genomic Sci. 2015 Aug 14;10:54. doi: 10.1186/s40793-015-0049-2 (PMC4572658; doi:10.1186/s40793-015-0049-2)
Supplement: Additional file 1: Table S1. — Associated MIGS record for Mesorhizobium loti CJ3Sym. (DOC 73 kb) [file 40793_2015_49_MOESM1_ESM.doc]

Associated MIGS Record

***Table S1.*** *Associated MIGS record for* [*Mesorhizobium loti*](http://dx.doi.org/10.1601/nm.1415) *CJ3Sym*

| **MIGS-ID** | field name | description |
| --- | --- | --- |
| **MIGS-1** | Submit to INSDC/Trace archives |  |
| **1.1** | PID |  |
| **1.2** | Trace Archive |  |
| **MIGS-2** | MIGS CHECK LIST TYPE |  |
| **MIGS-3** | Project Name | GEBA - Root Nodulating Bacteria |
| **MIGS-4** | Geographic Location | Dunedin, Otago, NZ |
| **4.1** | Latitude | -45.864179 |
| **4.2** | Longitude | 170.512551 |
| **4.3** | Depth |  |
| **4.4** | Altitude |  |
| **MIGS-5** | Time of Sample collection |  |
| **MIGS-6** | Habitat (EnvO) | Rocklands range in New Zealand |
| **6.1** | temperature | 28°C |
| **6.2** | pH | 4-10 |
| **6.3** | salinity |  |
| **6.4** | chlorophyll |  |
| **6.5** | conductivity |  |
|
| **6.6** | light intensity |  |
| **6.7** | dissolved organic carbon (DOC) |  |
| **6.8** | current |  |
| **6.9** | atmospheric data |  |
| **6.10** | density |  |
| **6.11** | alkalinity |  |
| **6.12** | dissolved oxygen |  |
| **6.13** | particulate organic carbon (POC) |  |
| **6.14** | phosphate |  |
| **6.15** | nitrate |  |
| **6.16** | sulfates |  |
| **6.17** | sulfides |  |
| **6.18** | primary production |  |
| **MIGS-7** | Subspecific genetic lineage |  |
| **MIGS-9** | Number of replicons |  |
| **MIGS-10** | Extrachromosomal elements |  |
| **MIGS-11** | Estimated Size | 7.6 Mbp |
| **MIGS-12** | Reference for biomaterial or Genome report |  |
| **MIGS-13** | Source material identifiers |  |
| **MIGS-14** | Known Pathogenicity | Non-pathogen |
|
| **MIGS-15** | Biotic Relationship | Symbiotic |
| **MIGS-16** | Specific Host | [*Lotus corniculatus*](http://www.theplantlist.org/tpl1.1/record/ild-7260) |
| **MIGS-17** | Host specificity or range (taxid) |  |
| **MIGS-18** | Health status of Host |  |
| **MIGS-19** | Trophic Level |  |
| **MIGS-22** | Relationship to Oxygen | Aerobe |
| **MIGS-23** | Isolation and Growth conditions | TY media [23], aerobe |
| **MIGS-27** | Nucleic acid preparation | CTAB |
| **MIGS-28** | Library construction | Illumina Std PE |
| **28.1** | Library size | 3,949 Mbp |
| **28.2** | Number of reads | 26,326,824 |
| **28.3** | vector |  |
| **MIGS-29** | Sequencing method | Illumina HiSeq 2000 |
| **MIGS-30** | Assembly |  |
| **30.1** | Assembly method | Velvet 1.1.04, Allpaths-LG V.r41043 |
| **30.2** | estimated error rate |  |
| **30.3** | method of calculation |  |
| **MIGS-31** | Finishing strategy |  |
| **31.1** | Status | High-quality Permanent Draft |
| **31.2** | coverage | 522x |
| **31.3** | contigs | 71 |
| **MIGS-32** | Relevant SOPs |  |
| **MIGS-33** | Relevant e-resources |  |
